# Deep Venous Thrombosis and Pulmonary Embolism Secondary to Mild Traumatic Injury in an Elderly Male With No Additional Risk Factors

**DOI:** 10.7759/cureus.28829

**Published:** 2022-09-06

**Authors:** Siddharth A Sheth, Charlotte DeGeorge, Andrew George, Thor S Stead, Rohan Mangal, Latha Ganti

**Affiliations:** 1 Emergency Medicine, University of Central Florida College of Medicine, Orlando, USA; 2 Department of Pathology and Laboratory Medicine, Division of Biology and Medicine, Brown University, Providence, USA; 3 Medicine, The Warren Alpert Medical School of Brown University, Providence, USA; 4 Medicine, University of Miami Miller School of Medicine, Miami, USA; 5 Emergency Medicine, HCA Florida Ocala Hospital, Ocala, USA; 6 Emergency Medicine, Envision Physician Services, Plantation, USA

**Keywords:** dvt risk factors, traumatic deep vein thrombosis, venous thromboembolism, pulmonary embolism, deep vein thrombosis

## Abstract

Venous thromboembolism (VTE) is a significant cardiovascular disease with a relatively high incidence rate, presenting a significant clinical burden. Its effective diagnosis and treatment are critical to the proper management of patients with the condition. Though there are several risk factors associated with VTE, advanced age itself presents as particularly significant, with age-related risk alone leading to high incidences of VTE in elderly patients even in the absence of other risk factors or relevant medical history. We present such a case of an elderly male patient with limited risk factors beyond advanced age who nonetheless presented with symptoms of deep-vein thrombosis (DVT) following a mild traumatic injury, and upon further inspection, was found to have a pulmonary embolism (PE). Proper precautions taken in assessing potential PE upon initial discovery of DVT and post-diagnostic management were critical in this patient's outcome.

## Introduction

Venous thrombosis refers to a blockage of a vein due to a thrombus, or blood clot. Deep vein thrombosis (DVT) is a type of venous thrombosis that occurs with the presence of a blood clot in the deep blood vessels. A thrombus originating in the legs can embolize to the lungs; this condition is known as a pulmonary embolism (PE). The conditions of DVT and PEs, separately or combined, can be considered venous thromboembolisms (VTEs) [1].

VTEs are the third most common cause of cardiovascular-related death in the United States, with an incidence rate of approximately 1-2 in 1000 [2]. Between 60,000 to 100,000 people die from PEs in the United States annually [3]. As a result, proactive and effective treatment of PEs is crucial to long-term patient survival. For any VTE, the most common treatment is an anticoagulative (AC) treatment, namely heparin and/or warfarin, which impedes the clotting of blood. Consequently, these drugs are also used to manage thrombosis risk. However, AC treatments are limited by a narrow therapeutic index, with potential adverse side effects including both hemorrhagic and, to a lesser extent, non-hemorrhagic side effects [4,5]. Warfarin, for instance, has been reported to carry an annual hemorrhagic side effect rate of 15-20% and a fatal hemorrhagic side effect rate of 1-3% [6]. Such complications necessitate careful management of both VTE risk and therapeutic risk of AC.

Risk factors for developing a DVT include increasing age (over 40 years), previous VTE, cardiac or respiratory failure, obesity, prolonged immobility, recent surgery, active cancer treatment, oral contraceptive pills, and a wide variety of inherited and acquired hematological conditions [7]. Although not more likely to be primarily diagnosed with DVT, men are more likely to experience recurring DVTs [1]. We present a case of a DVT and PE in an elderly male patient with a negative medical history of previous thrombotic events.

## Case presentation

A 79-year-old man presented to the emergency department (ED) with the chief complaint of significant pain and swelling in his left calf. Several days prior, the patient had bumped his leg while taking out the trash. His calf appeared to bruise and then heal; three days before his visit to the ED his left calf became rigid and provoked throbbing pain. 

The patient’s medical history was significant for diabetes, dyslipidemia, and hypertension. He did not have any major surgeries in the past nor taken any hormonal therapy. The patient reported no recent trauma, did not claim to lead a sedentary lifestyle, and denied a prior history of thrombotic events or a family history of blood clots. The patient did not report chest pain, shortness of breath, nausea, vomiting, or cough.

The patient's vital signs in the ED were: blood pressure 148/81 mmHg, a temperature of 97.9 °F, a pulse of 63 beats per minute, respirations at 16 breaths per minute, and saturation of 95% on room air. Physical examination revealed the muscle to be swollen as compared to the right calf and tender to palpation. There were no open wounds. An inspection of the ankle and foot showed no signs of swelling, erythema, deformity, edema, or injury of any kind.

**Figure 1:**
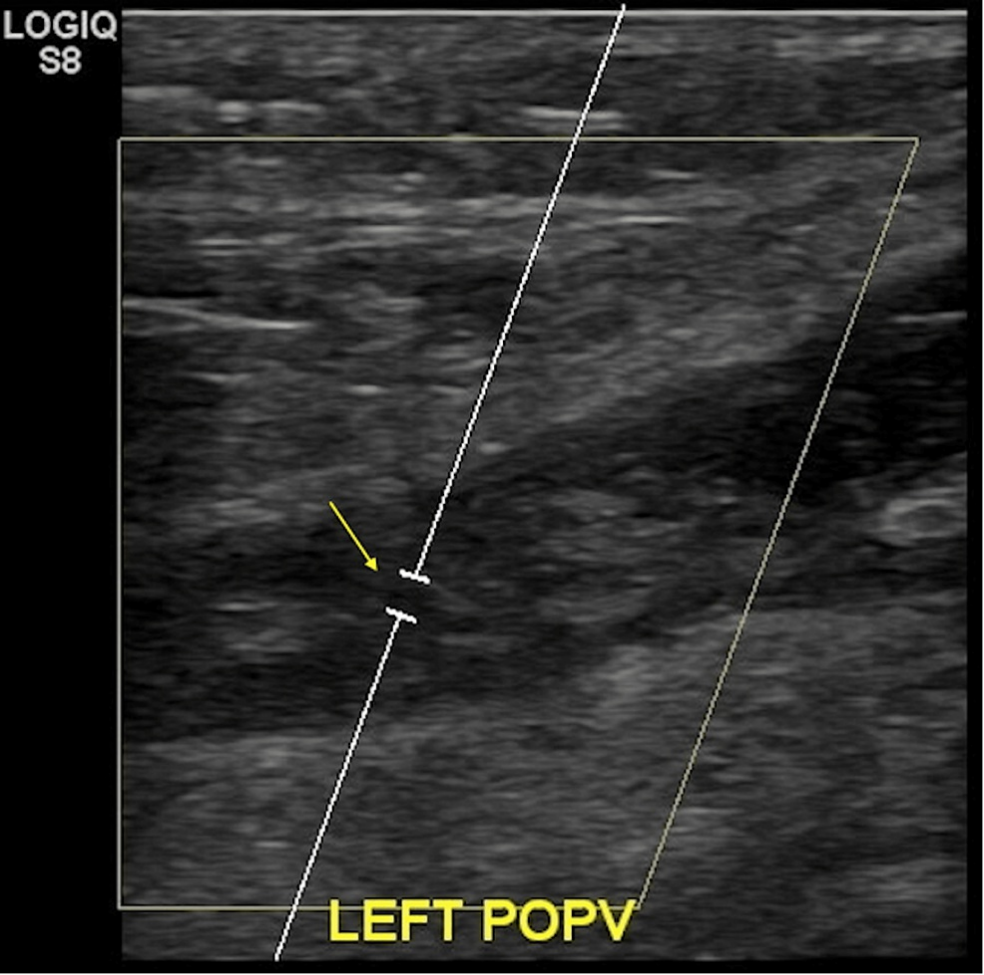
Imaging of patient’s lower left extremity reveals deep vein thrombosis as assessed by Doppler ultrasonography

**Figure 2:**
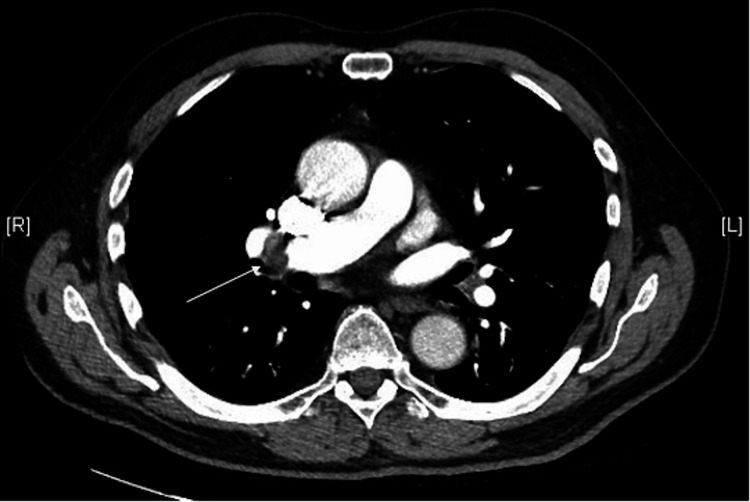
Image of the patient's pulmonary embolism as assessed by chest computed tomography

The patient’s laboratory results showed elevated glucose levels of 188 mg/dL, high hemoglobin A1C of 8.4%, high partial thromboplastin time of 40.5 seconds, and low red blood cell count of 3.99x10^6^/mm^3^. Doppler ultrasonography of the left lower extremity revealed DVT (Figure 1), and computed tomography angiography revealed right lower lobe pulmonary embolism with no evidence of right heart strain (Figure 2).

The patient was initially given one dose of apixaban 10 mg and then switched to a heparin drip after the PE was found. Intravenous (IV) heparin was continued to manage the patient through the course of 48 hours prior, after which the treatment plan switched to oral apixaban twice daily at 10 mg for seven days then reduced to 5 mg on the same schedule to continue anticoagulant therapy for six months. Upon stabilization of the patient's condition, the patient was discharged with an outpatient hematology follow-up scheduled.

## Discussion

The development of DVT and PE in this patient highlights the age-related risk of VTE even in absence of additional risk factors. Also notable is the discovery of PE upon chest CTA without accompanying signs or symptoms following initial identification of the DVT. Venous status, vascular injury, and hypercoagulability (Virchow's triad) all contribute to the formation of DVT. Commonly, clinical conditions associated with DVT are related to these three factors, such as pregnancy, surgery, immobility, malignancy, obesity, trauma, advancing age, and a history of DVT [8]. DVT has also been seen in association with coronavirus disease 2019 (COVID-19) and is associated with poorer outcomes on account of SARS-CoV-2 infection-related hypercoagulability [9]. Further, malignancies can similarly lead to hypercoagulability resulting in a greater risk of VTE [8]. Surgical risk factors also come with major orthopedic and neurovascular surgeries and increase significantly with ordered bed rest and immobility during recovery and extended hospitalization [7].

The patient, in this case, had previously undergone no recent surgeries, thus the cause of this DVT could not be attributed to risk factors associated with prior surgery. Although this patient was significant for age-related risk of DVT, he was not obese, did not smoke, had no relevant family history, and kept a relatively active lifestyle.

The patient experienced a trauma-induced DVT. Interestingly, however, this patient experienced only relatively minor trauma. This relatively low magnitude of traumatic injury (the patient's collision with a garbage can) and the fluctuation of symptoms suggest cause for an in-depth analysis of potential underlying factors.

The treatment for most cases of venous thromboembolism is anticoagulation. The duration of oral anticoagulation is dependent on the cause. A spontaneous case may necessitate life-long anticoagulation, whereas a secondary cause may only require three-six months. In some severe cases such as phlegmasia cerulea dolens, thrombolysis and thrombectomy are considerations. Submassive and massive PEs may also require thrombolysis or thrombectomy. 

## Conclusions

Given the patient’s limited risk factors, the formation of a DVT and subsequent PE is surprising. This case highlights the effect of age as a primary risk factor on vascular structural integrity and serves as a reminder of increased susceptibility to even seemingly minor traumatic injuries that may result in life-threatening thrombotic events. Consequently, it is important to consider VTE in elderly patients with similar clinical presentation as a possible diagnosis, particularly screening for PE even in absence of obvious symptoms.
